# A new development of non-local image denoising using fixed-point iteration for non-convex *ℓ_p_* sparse optimization

**DOI:** 10.1371/journal.pone.0208503

**Published:** 2018-12-12

**Authors:** Shuting Cai, Kun Liu, Ming Yang, Jianliang Tang, Xiaoming Xiong, Mingqing Xiao

**Affiliations:** 1 School of Automation, Guangdong University of Technology, Guangzhou, China; 2 Department of Computer Science, Southern Illinois University-Carbondale, Carbondale, IL, United States of America; 3 Department of Mathematics, Southern Illinois University-Carbondale, Carbondale, IL, United States of America; 4 College of Mathematics and Statistics, Shenzhen University, Shenzhen, Guangdong Province, China; INSERM, FRANCE

## Abstract

We proposed a new efficient image denoising scheme, which mainly leads to four important contributions whose approaches are different from existing ones. The first is to show the equivalence between the group-based sparse representation and the Schatten-*p* norm minimization problem, so that the sparsity of the coefficients for each group can be measured by estimating the underlying singular values. The second is that we construct the proximal operator for sparse optimization in *ℓ*_*p*_ space with *p* ∈ (0, 1] by using fixed-point iteration and obtained a new solution of Schatten-*p* norm minimization problem, which is more rigorous and accurate than current available results. The third is that we analyze the suitable setting of power *p* for each noise level *σ* = 20, 30, 50, 60, 75, 100, respectively. We find that the optimal value of *p* is inversely proportional to the noise level except for high level of noise, where the best values of *p* are 1 and 0.95, when the noise levels are respectively 75 and 100. Last we measure the structural similarity between two image patches and extends previous deterministic annealing-based solution to sparsity optimization problem through incorporating the idea of dictionary learning. Experimental results demonstrate that for every given noise level, the proposed Spatially Adaptive Fixed Point Iteration (SAFPI) algorithm attains the best denoising performance on the value of Peak Signal-to-Noise Ratio (PSNR) and structure similarity (SSIM), being able to retain the image structure information, which outperforms many state-of-the-art denoising methods such as Block-matching and 3D filtering (BM3D), Weighted Nuclear Norm Minimization (WNNM) and Weighted Schatten *p*-Norm Minimization (WSNM).

## 1 Introduction

Images are generally contaminated by noise during acquisition, transmission and compression and real-life images are often degraded with mixed noise and it is hard to identify the type and model the noise [1–8]. Images with high resolutions are desirable in many applications, e.g., object recognition, image classification, and image segmentation in medical and biological science. As an essential low-level image processing procedure, image denoising has been studied extensively and belong to a special type of classical inverse problems. The general observation with additive noise can be modeled as **Y** = **X** + **N**, where **Y** is the noisy observation, and **X** and **N** present the original image and white Gaussian noise, respectively. Though a plethora of noise removal techniques have appeared in recent years, for example, Convolutional Neural Network (CNN) [9, 10] have proved very promising on denoising tasks for which large training sets are available, but when the training data are scarce, their performance suffers from overfitting. Therefore image denoising for real-life noise still remains an important challenge in order to recover the images with high quality [11].

Image denoising problem is in general ill-posed and it requires appropriate regularization. Over the past few decades, numerous image denoising methods have been developed [12]. This is usually achieved by minimizing a suitable energy functional that characterizes a trade-off between data-fidelity and regularity. Frobenius norm is often employed to measure the data fitting loss for additive Gaussian noise.

Sparse signal representation describes a signal that can be approximated as a linear combination of as few as possible atoms from a given dictionary. Recently, Elad [13] showed that sparse overcomplete representation approach is quite effective in denoising images, supported by recent study that better denoising performance can be achieved by using a variant of sparse coding methods [14, 15]. In order to promote sparsity more extensively than convex regularization, it is also standard practice to employ non-convex optimization [16].

In image denoising, following [17], each noise patch **y**_*i*_ is extracted from the noisy image **Y**. In order to better exploit group sparsity, we group a set of similar patches $Y=\left[y_{1},y_{2},\ldots ,y_{n}\right]\in R^{m\times n}$. Thus, denoising problem becomes the recovery problem of **x**_*i*_ from **y**_*i*_. Now let us consider the group sparsity defined by a group norm ||**A**||_*p*,2_:

$$\begin{array}{c} \left(D,A\right)=\arg\min_{D,A}\frac{1}{2}||Y-{\text{DA}||}_{F}^{2}+{\lambda ||A||}_{p,2}^{p},\;0<p\leq 1, \end{array}$$ (1)

where $A=\left[\alpha^{1},\alpha^{2},\ldots ,\alpha^{n}\right]\in R^{m\times n}$ is related to image patches by **X** = **DA**. We note that the group norm (quadratic symmetric gauge function, see 2.4.2 of [18])||⋅||_*p*,2_ is defined by

$$\begin{array}{c} {\left||A|\right|}_{p,2}≜∥(||\alpha^{1}{\left|\right|}_{2},\ldots ,||\alpha^{n}{\left|\right|}_{2}{)∥}_{p}, \end{array}$$

where *α*^*i*^ = [*α*_*i*,1_, …, *α*_*i*,*m*_]^*T*^ denotes the *i*^*th*^ column of matrix **A** in $R^{m\times n}$. In recent years, many research is devoted to address the group sparse optimization problem (1), aiming at the improvement of efficiency and accuracy (e.g., see survey paper [16] and references therein).

Once all group sparse codes **A** are achieved, the latent clean image **X** can be reconstructed as **X** = **DA** by standard approach(see Theorem 1 and 2 in [19]).

The main contributions of this paper are illustrated as follows:

1. We unify the group-based sparse coding in [20] and the Schatten-*p* norm minimization problem in [21] by proving their mathematical equivalence.
2. A fixed-point iteration scheme is developed for sparse optimization in *ℓ*_*p*_ space with *p* ∈ (0, 1] by using proximal operator and we a new solution to Schatten-*p* norm minimization problem is obtained, which appears to be more accurate and rigorous than [21].
3. Regarding to image denoising, we find that the optimal value of *p* is inversely proportional to the noise level except for high level noise, where the best values of *p* are 1 and 0.95, when the noise levels are 75 and 100, respectively.
4. The proposed Spatially Adaptive Fixed Point Iteration (SAFPI) algorithm attains the best denoising performance on the value of PSNR and SSIM, being able to retain the image structure information, which outperforms many state-of-the-art denoising methods such as BM3D, WNNM and WSNM.

The rest of the paper is organized as follows. In Section 2.1, we prove the equivalence of group-based sparse coding and the Schatten-*p* norm minimization problem and propose a new solution to Schatten-*p* norm minimization problem. A fixed point iteration for solving sparse optimization in *ℓ*_*p*_ space with *p* ∈ (0, 1] is formulated and discussed. In Section 2.2, we establish an image denoising scheme using nonlocal self-similarity and Schatten-*p* norm minimization. In Section 3, based on the new developed Spatially Adaptive Fixed Point Iteration (SAFPI) algorithm, we present the experimental results using a set of standard benchmark images. And the comparison with several existing methods are also provided to demonstrate our improvement. Finally, the paper ends with concluding remarks.

## 2 Materials and methods

### 2.1 Proximal operator for Schatten-p norm minimization

#### 2.1.1 Background

Consider a matrix $Y\in R^{m\times n}$, then **Y**^*T*^ **Y** is a positive semidefinite matrix. The eigenvalues of **Y**^*T*^ **Y** are called the singular values of **Y**, denoted by *σ*_1_(**Y**), …, *σ*_min{*m*,*n*}_(**Y**) in decreasing order (see page 246 of [22]). Let *r* = **rank**(**Y**), it is clear that

$$\begin{array}{c} \sigma_{r+1}\left(Y\right)=0,\ldots ,\sigma_{\min\{m,n\}}\left(Y\right)=0. \end{array}$$

The matrix **Y** also has the following Singular Value Decomposition (SVD) **Y** = **U**Σ**V**^*T*^, where U∈O(m),V∈O(n) (O is the set of orthogonal matrices) and *σ*_*n*_ is an *m*×*n* diagonal matrix with diagonal entries *σ*_1_(**Y**), …, *σ*_min{*m*,*n*}_(**Y**). We introduce the Schatten-*p* norm (0 < *p* < ∞) of **Y**, which is defined as

$$\begin{array}{c} {\left|\left|Y\right|\right|}_{S_{p}}={\left(\sum_{i=1}^{\text{min}\left\{m,n\right\}}\sigma_{i}^{p}\left(Y\right)\right)}^{1/p}. \end{array}$$

Special cases of the Schatten-*p* norm include the nuclear norm (*p* = 1) and the Frobenius norm (*p* = 2).

Next we analyze the relationship between group-based sparse coding and the Schatten-*p* norm minimization problem, which improves Theorem 2 in [23]. But our approach is based on the “symmetry” technique (similar to [17] for other purpose), which is essentially different from [23].

**Theorem 1** *The group-based sparse coding problem* (1) *is equivalent to a Schatten-p norm minimization problem*.

Eqs (12), (13) and (14) imply that any operation designated for sparse coefficient vector *α*’s can be conveniently implemented with singular values of **X** (only differs by a constant scalar).

The Schatten-*p* norm (0 < *p* ≤ 1) has been widely used to replace the nuclear norm for better approximating the rank function. There are extensive study for the Schatten-*p* norm optimization problem (14) in literature [24, 25]. Note that the main difference between group sparse coding and the Schatten-*p* norm minimization problem is that group sparse coding has a dictionary learning operator while the Schatten-*p* norm minimization problem does not involve such operation.

#### 2.1.2 Computation of proximal mapping using fixed point iterative method

Now let us recall the definition of proximal mapping.

**Definition 2** *The proximal mapping of a mapping* Θ:R↦R is

$$\begin{array}{c} {\text{Prox}}_{\Theta }\left(x\right)=\arg\min_{w}\left\{{\left(w-x\right)}^{2}+2\lambda \Theta \left(w\right)\right\}. \end{array}$$

*The proximal mapping of* ${\left||Y|\right|}_{S_{p}}^{p}$ *is defined as*:

$$\begin{array}{c} {\text{Prox}}_{\lambda }∥\cdot {∥}_{p}\left(Y\right)=\arg\min_{X}\frac{1}{2}||Y-{X||}_{F}^{2}+{\lambda ||X||}_{S_{p}}^{p}. \end{array}$$ (2)

And we have the following celebrated theorem:

**Theorem 3** *[Theorem 1 of* [26]*] If matrix* $Y\in R^{m\times n}$ *has the following Singular Value Decomposition (SVD)* **Y** = **U**Σ**V**^*T*^, *where* U∈O(m),V∈O(n) *and σ*_*n*_ *is an m × n diagonal matrix with diagonal entries σ*_1_(**Y**), …, *σ*_min{*m*,*n*}_(**Y**). *Then we have in* Eq (2)

$$\begin{array}{c} \hat{X}={\text{Prox}}_{\lambda }∥\cdot {∥}_{p}\left(Y\right)=U\text{diag}\left(\sigma_{i}\left(\hat{X}\right)\right)V^{T}, \end{array}$$ (3)

*where* $\sigma_{i}\left(\hat{X}\right)$ *is defined as the scalar proximal mapping in* (2):

$$\begin{array}{c} \sigma_{i}\left(\hat{X}\right)={\text{Prox}}_{\lambda }∥\cdot {∥}_{p}\left(\sigma_{i}\left(Y\right)\right)=\arg\min_{\sigma_{X}\geq 0}\frac{{\left(\sigma_{X}-\sigma_{i}\left(Y\right)\right)}^{2}}{2}+\lambda \sigma_{X}^{p}. \end{array}$$ (4)

In order to be transparent for our proposed approach to solve Eq (4), we recall two important concepts in convex optimization next.

**Definition 4** *(see Chapter 2 p 82 of* [27]) *Let* $R^{n}$ *be paired by a bilinear functional (inner product)* 〈,〉 *and let* $f:R^{n}\mapsto R$ *be any extended real-valued function on* $R^{n}$. Then the function f* *on* $R^{n}$ *defined by*

$$\begin{array}{c} f^{*}\left(y\right)=\min_{x}f\left(x\right)-\left\langle x,y\right\rangle ,\;y\in R^{n} \end{array}$$

is called the Fenchel conjugate of f (with respect to the given pairing). Note that f* *is always a closed convex function, regardless of the structure of f*.

**Definition 5** *Given the proper convex function* $f:R^{n}\mapsto \left(-\infty ,+\infty \right]$, *the subdifferential of such a function is the (generally multivalued) mapping* $\partial f:R^{n}\mapsto R^{n*}$ *defined by*

$$\begin{array}{c} \partial f\left(x\right)=\{x^{*}\in R^{n*}|f\left(z\right)\geq f\left(x\right)+\left\langle x^{*},z-x\right\rangle ,\;z\in R^{n}\}. \end{array}$$

The elements x* ∈ ∂*f*(*x*) *are called subgradients of f at x. Actually, same definition works for nonconvex f (however, subgradient need not exist)*.

A point $x\in R^{n}$ is a minimizer of a function *f* (not necessarily convex) over $R^{n}$ if and only if *f* is subdifferentiable at *x* and 0 ∈ ∂*f*(*x*).

**Lemma 6**

$$\begin{array}{c} {\text{Prox}}_{\lambda }∥\cdot {∥}_{p}\left(x\right)=\arg\min_{w}{\{\left(w-x\right)}^{2}+{2\lambda |w|}^{p},0<p\leq 1\} \end{array}$$

*is closed and convex, but it has no close form solution for general p*.

If *p* = 1, it is well-known that the function *ϕ*(*w*) = |*w*| is not differentiable but still convex, and can be described by a subgradient (see Section 2.3 of [28]) as ∂*ϕ*(*w*) = *sign*(*w*) and from Lemma 6, we have

$$\begin{array}{c} {\text{Prox}}_{\lambda }∥\cdot {∥}_{1}\left(x\right)={\left(I+\lambda \partial \phi \right)}^{-1}\left(x\right)=\max\left\{|x|-\lambda ,0\right\}. \end{array}$$

Furthermore, we can obtain the following theorem, which improves Theorem 1 in [29] and Theorem 1 in [19] using fixed-point iteration (see Chapter 1 of [30] for details).

**Definition 7** *Given a function* g:[a,b]↦R, *find ξ* ∈ [*a*, *b*] *such that ξ* = *g*(*ξ*). *If such ξ exists, it will be called a fixed point of g and it could be computed by the following algorithm: ξ*^(*n*)^ = *g*(*ξ*^(*n*−1)^), *n* ≥ 1. *And g is said to be a contraction on* [*a*, *b*] *if there exists a constant L such that* 0 < *L* < 1 *and* |*g*(*x*) − *g*(*y*)| < *L*|*x* − *y*| *for any x, y* ∈ [*a*, *b*].

**Theorem 8** *Let us denote*

$$\begin{array}{c} {ℵ}_{\lambda ,p}=\max\left\{\frac{2-p}{2(1-p)}{\left(2\lambda \left(1-p\right)\right)}^{\frac{1}{2-p}},\frac{1}{2}{\left(\lambda p\left(1-p\right)\right)}^{\frac{1}{2-p}}+\lambda {\left(\lambda p\left(1-p\right)\right)}^{\frac{p-1}{2-p}}\right\}, \end{array}$$

*then for* 0 < *p* < 1, *we have*

$$\begin{array}{ll} Prox_{\lambda }∥\cdot {∥}_{p}\left(x\right) & =\text{arg}\;\underset{w\geq 0}{\text{min}}\{{\left(w-x\right)}^{2}+2\lambda {\left|w\right|}^{p}\}\\ & =\{\begin{array}{ll} 0 & \left|x\right|\leq {ℵ}_{\lambda ,p}\\ fixed-point\;of\;the\;contraction\;w\mapsto \left|x\right|-\lambda p\frac{w}{{\left|w\right|}^{2-p}} & \left|x\right|\geq {ℵ}_{\lambda ,p}. \end{array} \end{array}$$

### 2.2 Spatially Adaptive Fixed Point Iteration (SAFPI) denoising algorithm

In [20, 21], the authors proposed a group sparse representation framework and a Schatten-*p* norm minimization framework for image denoising. In Theorem 1, we have shown these two approaches are equivallent. From combining Theorem 3 and Theorem 8, we obtained a fixed point iteration solution of Eq (14) in Theorem 1, which is more rigorous than [20, 21].

After grouping a set of similar patches $Y=\left[y_{1},y_{2},\ldots ,y_{n}\right]\in R^{m\times n}$, the denoising problem becomes the recovery problem of **x**_*i*_ from **y**_*i*_. And as was shown in Theorem 1, the Schatten-*p* norm minimization problem (14) converts the denoising problem to recover the low-rank matrix **X** from the non-low-rank matrix **Y**, and thus filtering out the noise of the structure set. And the second identity in Eq (14) can be solved using Theorem 3 and Theorem 8.

Wavelet-based image denoising assumes that the wavelet coefficients obey the Laplace distribution, and the threshold method is used to filter the noise in the image. The prior distribution of the block matrix singular values can also approximate the Laplace distribution in space. The parameter λ for each group that balances the fidelity term and the regularization term should be adaptively determined for better denoising performance. Using the Spatial Adaptive Laplacian Transcendental as appeared in [17, 31], the threshold parameter can be set to $\lambda_{i}=\frac{2\sqrt{2}\sigma_{w}^{2}}{\sigma_{i}},$ where *σ*_*i*_ denotes the locally estimated variance at the position *i*. Now the second identity in Eq (14) becomes

$$\begin{array}{c} {\text{Prox}}_{\lambda_{i}}∥\cdot {∥}_{p}\left(Y\right)=\arg\min_{X}\frac{1}{2}||Y-{X||}_{F}^{2}+\frac{2\sqrt{2}\sigma_{w}^{2}}{\sigma_{i}}{\left||X|\right|}_{S_{p}}^{p}. \end{array}$$ (5)

From Eq (4), if **Y** has singular value decomposition **Y** = **U**Σ**V**^*T*^, we have $\hat{X}=U\hat{\Sigma }V^{T}$, where $\hat{\Sigma }=\text{diag}\left\{{\hat{\varepsilon }}_{1},\ldots ,{\hat{\varepsilon }}_{\min\{m,n\}}\right\}$. And ${\hat{\varepsilon }}_{i}$ can be computed using Theorem 3 and Theorem 8.

Recently some developed iterative regularization techniques in [17] offers an alternative approach toward spatial adaptation. The basic idea of iterative regularization is to add filtered noise back to the denoised image i.e.,

$$\begin{array}{c} y^{\left(k+1\right)}={\hat{x}}^{\left(k\right)}+\delta \left(y-{\hat{x}}^{\left(k\right)}\right) \end{array}$$ (6)

where *k* denotes the iteration number and *δ* is a relaxation parameter. Besides, we can execute the above denoising procedures for better results after several iterations. In the *k* + 1-th iteration, the iterative regularization strategy in [17] is used to update the estimation of noise variance. Then the standard deviation of noise in *k* + 1-th iteration is adjusted as

$$\begin{array}{c} {\hat{\sigma }}_{\omega }^{\left(k+1\right)}=\gamma \sqrt{\sigma_{\omega }^{2}-||y-y^{\left(k\right)}{\left|\right|}_{2}^{2}} \end{array}$$ (7)

where *γ* is a scaling factor controlling the re-estimation of noise variance and the local estimated variance at the *i*-th position is

$$\begin{array}{c} {\hat{\sigma }}_{i}^{\left(k+1\right)}=\sqrt{\text{max}\left({\left(\chi_{i}^{\left(k\right)}\right)}^{2}/n-{\left({\hat{\sigma }}_{\omega }^{\left(k\right)}\right)}^{2},0\right)}. \end{array}$$ (8)

where *χ*_*i*_ is the *i*-th singular value of image **y**.

The higher the structural similarity of the blocks in the structure group is, the more correlative the column vectors in the block matrix will be, which means that it has a low rank property corresponding to the noise-free matrix. The information is mainly concentrated in those largest singular values. During the proximal operation, selecting the appropriate threshold parameter for those with larger singular value makes the processed singular value closer to the noise-free singular value, which can well preserve the useful information in the image while filtering out the noises.

Therefore, choosing blocks with more similar structure will help to improve the image denoising effect. There are many commonly used similarity measures such as Euclidean distance, cosine angle, and correlation coefficient. The traditional block similarity measure function have some shortcomings in measuring the similarity between blocks. Euclidean distance simply calculate the difference between the pixel gray value of the blocks, and then add up as a standard measure of the degree of similarity. Although this method is simple and easy to implement, it only treats the blocks as isolated pixels and neglects the statistical relevance between local pixels, which leads to the inaccuracy of similarity measure. This is because the blocks are not in an Euclidean space. There is a very strong correlation between the pixels in the block. The local pixel correlation carries important structural information of the blocks.

In order to solve this problem, Structural SIMilarity (SSIM) index [32, 33] is often used to evaluate the image quality. SSIM is defined as $\left(2\mu_{X}\mu_{\hat{X}}+c_{1}\right)\left(\sigma_{X,\hat{X}}+c_{2}\right)/\left[\left(\mu_{X}^{2}\mu_{\hat{X}}^{2}+c_{1}\right)\left(\sigma_{X}^{2}\sigma_{\hat{X}}^{2}+c_{2}\right)\right]$, where $\mu_{X},\mu_{\hat{X}}$, $\sigma_{X}^{2},\sigma_{\hat{X}}^{2}$, and $\sigma_{X,\hat{X}}$ denote the average of *X*, the average of $\hat{X}$, the variance of *X* and the variance of $\hat{X}$, respectively. *c*_1_ and *c*_2_ are two variables to stabilize the division with weak denominator.

A detailed step-by-step description of Spatially Adaptive Fixed Point Iteration (SAFPI) denoising algorithm is given by Algorithm 1

**Algorithm 1** Image Denoising via SAFPI Algorithm

**Require:** Initialization: $\hat{x}=y$;

 Iterate on *i* = 1,2, …, *iter*

1. Iterative regularization: $y^{\left(k+1\right)}={\hat{x}}^{\left(k\right)}+\delta \left(y-{\hat{x}}^{\left(k\right)}\right)$ and compute its variance $\sigma_{\omega }^{\left(k+1\right)}$;
2. Divide **y**^(*k*+1)^ into several blocks, the SSIM is used to classify the blocks with structural similarities into one structural group **Y**_*i*_;
3. Noise variance update: re-estimate *σ*_*ω*_ from **y**^(*k*+1)^ via ${\hat{\sigma }}_{\omega }^{\left(k+1\right)}=\gamma \sqrt{\sigma_{\omega }^{2}-||y-y^{\left(k+1\right)}{\left|\right|}_{2}^{2}}$;
4. SVD for each noisy data matrix **Y**_*i*_: (**U**_*i*_, Σ_*i*_, **V**_*i*_) = *SVD*(**Y**_*i*_), where Σ_*i*_ = **diag**{*ε*_1_, …, *ε*_min{*m*,*n*}_};
5. Thresholds update: compute λ_*i*_ using $\lambda_{i}=\frac{2\sqrt{2}\sigma_{\omega }^{2}}{\sigma_{i}^{\left(1/p\right)}}$ and ${\hat{\sigma }}_{i}=\sqrt{\max(\Sigma_{i}^{2}/n-\sigma_{\omega }^{2},0)}$;
6. Compute
  $$\begin{array}{c} {ℵ}_{\lambda_{i},p}=\max\left\{\frac{2-p}{2(1-p)}{\left(2\lambda_{i}\left(1-p\right)\right)}^{\frac{1}{2-p}},\frac{1}{2}{\left(\lambda_{i}p\left(1-p\right)\right)}^{\frac{1}{2-p}}+\lambda_{i}{\left(\lambda_{i}p\left(1-p\right)\right)}^{\frac{p-1}{2-p}}\right\}, \end{array}$$
7. Application of proximal operator: Updating the *ε*_*i*_ value by using the follow formula
  $$\begin{array}{c} \varepsilon_{i}=\{\begin{array}{cc} 0 & |\varepsilon_{i}|\leq {ℵ}_{\lambda_{i},p}\\ |\varepsilon_{i}|-\lambda_{i}p(|\varepsilon_{i}|-\lambda_{i}p|\varepsilon_{i}{{|}^{p-1})}^{p-1} & |\varepsilon_{i}|\geq {ℵ}_{\lambda_{i},p}, \end{array} \end{array}$$
  with computed λ_*i*_ and *ε*_*i*_ from step 5 and 4;
8. Image update: obtain an improved denoised image ${\hat{x}}^{\left(k\right)}$ by weighted averaging all denoised patches ${\hat{X}}_{i}=U_{i}{\hat{\Sigma }}_{i}V_{i}^{T}$, where ${\hat{\Sigma }}_{i}=\text{diag}\left\{\varepsilon_{1},\ldots ,\varepsilon_{\min\{m,n\}}\right\}$;

 Output: ${\hat{x}}^{\left(k\right)}$.

## 3 Results and discussion

**Fig 1:**
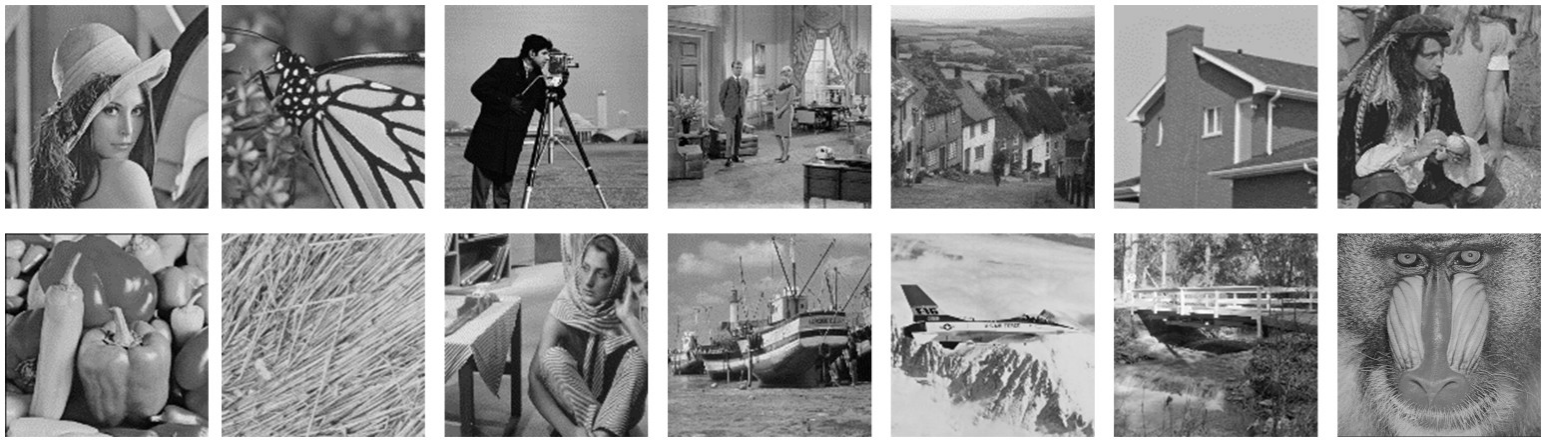
The 14 test images for image denoising.

In recent years, many denoising algorithms have been developed and the adaptive image removal algorithms [34–36] is a hot trend in signal and image denoising. To demonstrate the effectiveness of the proposed denoising algorithm, in this section, we compared the denoising performance with recently proposed state-of-the-art denoising methods, such as BM3D [37], WNNM [38], WSNM [21], Expected Patch Log-likelihood (EPLL) [39], Spatially Adaptive Iterative Singular-value Thresholding (SAIST) [17], Patch-Based Near-Optimal image denoising (PBNO) [40], Global Image Denoising (GID) [41], iterative denoising system based on Wiener filtering (WIENER) [34], and Linear Complex Diffusion Process (LCDP) [35]. We have used some well known images that are commonly used in the literature such as [17, 21, 38, 42]. We added noise to them, and test the proposed denoising algorithm with different power *p* under different noise levels. The experimental images are shown in Fig 1.

There are several image quality evaluation indicators measuring success of denoising such as kurtosis, low signal-to-noise-ratio(SNR). Low kurtosis indicate superior performance and it is defined as $k\left(X\right)=C_{4}\left(X\right)/C_{2}^{2}$ [43], where *C*_*k*_(.) is the *k*-th cumulant function. In our work, we evaluated the performance with three criterion: Structure Similarity Index (SSIM), kurtosis and Peak Signal-to-Noise Ratio (PSNR) which defined as $10\log_{10}\frac{M^{2}}{MSE}$, where *M* denotes the maximum intensity of the underlying image and $MSE=\frac{1}{n_{1}\times n_{2}}\sum_{i=1}^{n_{1}}\sum_{j=1}^{n_{2}}{\left(X_{i,j}-{\hat{X}}_{i,j}\right)}^{2}$ is the mean squared error between the denoised image $\hat{X}$ and the noiseless image *X*. All the experiments were carried out on Matlab (R2016a) of a PC with Intel(R) Xeon(R) CPU *E*5 − 1630 *V*4@3.7*GHz* and 32GB RAM.

### 3.1 Analysis of over-shrinkage problem and optimal power *p*

**Fig 2:**
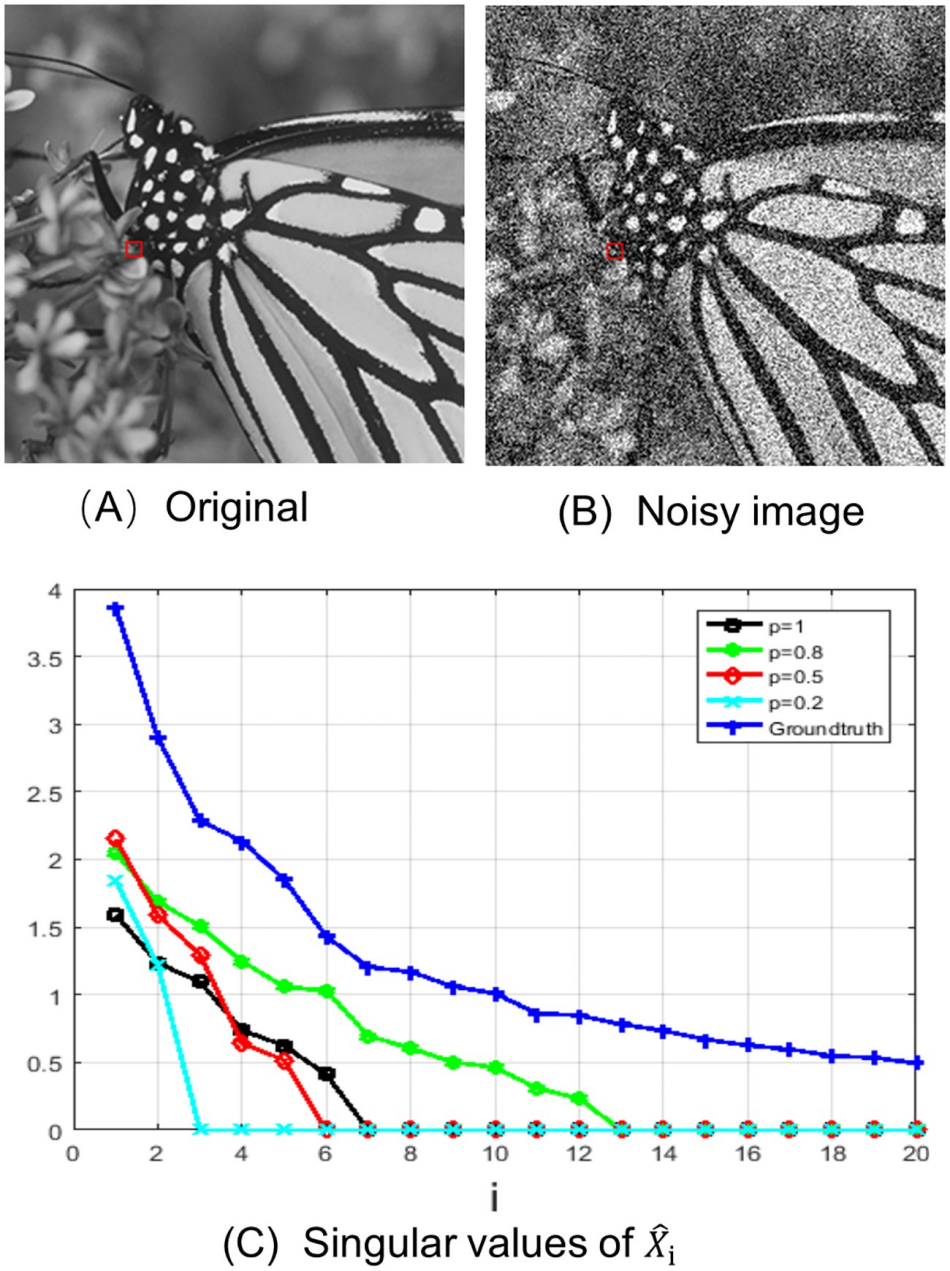
Illustration of the over-shrinkage problem with the value of power *p*. (A) Original image. (B) Noisy image with *σ*_*n*_ = 50. (C) Singular values of ${\hat{X}}_{i}$.

Firstly, we noticed that not all values of power *p* applied well to the proposed Spatially Adaptive Fixed Point Iteration (SAFPI) algorithm. It would conduct an approximation deviation with the solved singular values and produce excessive contraction, when the value of *p* is not suitable. As shown in Fig 2, we tested SAFPI to process low rank approximation on the red patch in Fig 2B with the noise level be 50, which is randomly marked from “Monarch” Fig 2A. In Fig 2C, $\left\{\sigma_{i}^{\left(p\right)}\right\}$ represents the singular values of the denoised similar patches with different power *p*. The ground-truth line (denoted by blue line) is the singular value connection line for the similar blocks of the noiseless red patch in Fig 2A. Now we can see that $\left\{\sigma_{i}^{\left(0.8\right)}\right\}$ (shown on green line) is more close to the ground-truth line. This means that the other $\left\{\sigma_{i}^{\left(p\right)}\right\}$’s (denoted by black, red, blue lines) conducted a serious over-shrinkage problem. In this case, setting *p* = 1 as in WNNM in denoising will lead to bad processing results. So the advantage of SAFPI algorithm is to overcome the over-shrinkage problem, in case we can find the optimal value of power *p*.

**Fig 3:**
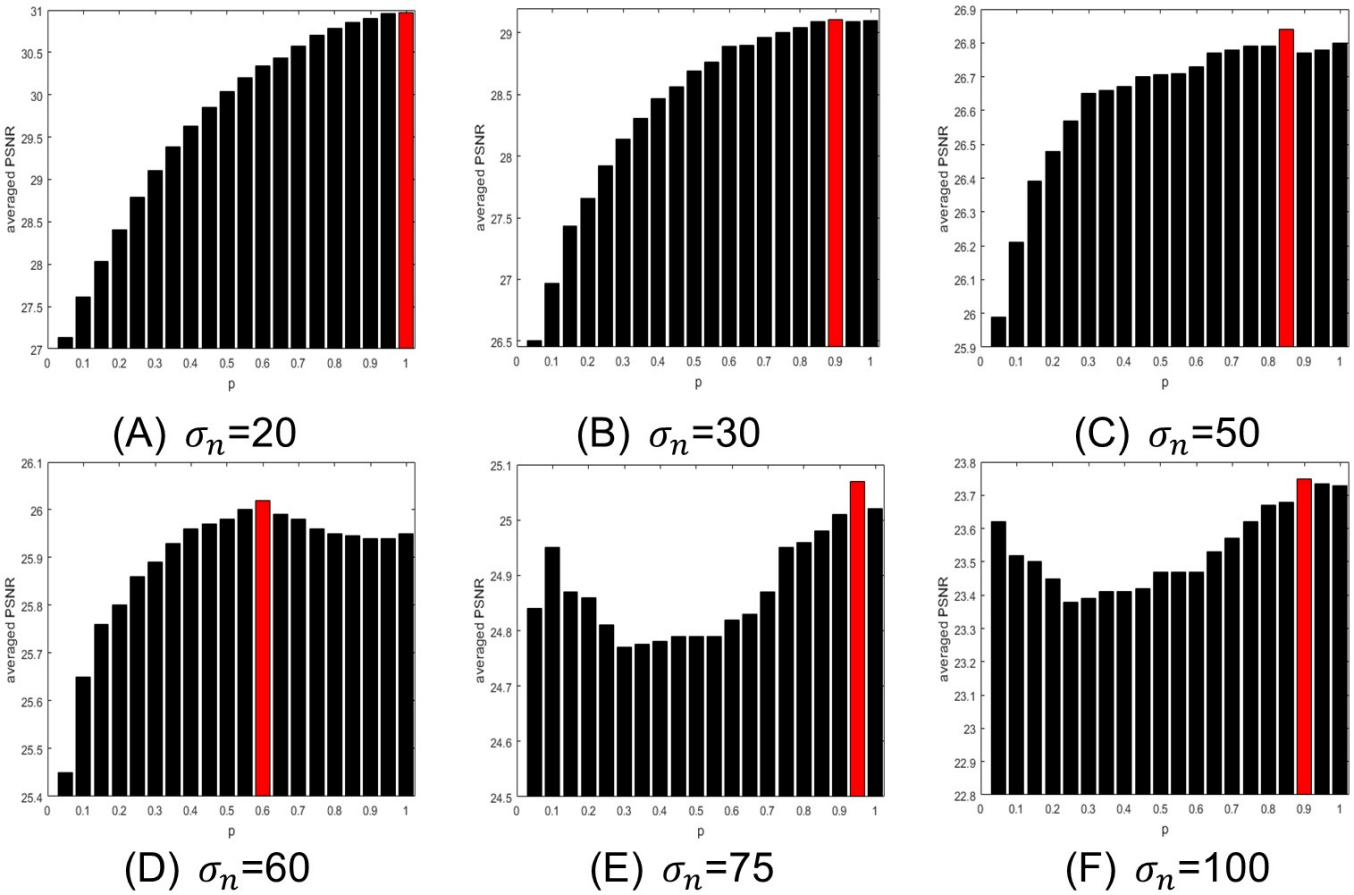
The influence of changing *p* upon denoised results under different noise levels. (A) *σ*_*n*_ = 20 (B) *σ*_*n*_ = 30 (C) *σ*_*n*_ = 50 (D) *σ*_*n*_ = 60 (E) *σ*_*n*_ = 75 (F) *σ*_*n*_ = 100.

Secondly, in order to find the optimal values of *p* under different noise levels for SAFPI algorithm, we randomly chose 10 test images in Fig 1 for our experiments and set the values of power *p* to be from 0.05 to 1 with an interval of 0.05. The zero mean additive white Gaussian noise levels were set to be *σ*_*n*_ = {20, 30, 50, 60, 75, 100}, and the other parameters were the same as WSNM [21]. The results are shown in Fig 3, the horizontal coordinate denotes the different values of *p* and the vertical coordinate represents the average value of PSNR under given noise level. And the red dots are the optimal points for each given noise level.

We can see that the best values of power *p* are 1.0, 0.90, 0.85 and 0.6, when the noise levels are low or medium 20, 30, 50 and 60, respectively. While handling very high noise levels 75, 100, the average PSNR values decrease firstly and then increase, the best values of *p* are 0.95 and 0.9 respectively. To sum up, we find that the optimal value of *p* is inversely proportional to the noise level except for high level of noise, where the best values of *p* are 1 and 0.95. And then we applied the best empirical values for the next experiments.

### 3.2 Performance comparison with different methods

We set *p* = {1.0, 0.9, 0.85, 0.6, 0.95, 0.9} for *σ* = {20, 30, 50, 60, 75, 100} in our proposed SAFPI algorithm. And then we compared the performance with seven standard algorithms (BM3D, WNNM, WSNM, EPLL, SAIST, PBNO, GID, WIENER, LCDP) from 13 widely used images from Fig 1. The results (thanks to the source codes provided by the authors) are in Tables 1, 2, 3, 4, 5 and 6. It can be seen from Table 7 that our algorithm always obtains the best average values of PSNR under different noise levels. The proposed approach achieves 0.3dB to 0.51dB improvement on average over the BM3D, when the noise levels are between 20 and 100. It also achieves 0.02dB, 0.06dB and 0.14dB improvement on average over the WSNM, when the noise levels are 30, 50 and 100, respectively. And our average values of SSIM are the best when the noise levels are 20, 30, 50 and 60. To sum up, for every given low and medium noise level, our algorithm attains the best denoising performance on the values of SSIM and PSNR for all noise levels. This leads to a better image denoising performance and high robustness to noise strength in comparison to several existing denosing algorithms.

**Table 1:** Denoising results of different algorithms for given noise level *σ*_*n*_ = 20.

	PSNR/*σ*_*n*_ = 20										SSIM/*σ*_*n*_ = 20									
Image	SAFPI	BM3D	PBNO	EPLL	GID	SAIST	WNNM	WSNM	WIENER	LCDP	SAFPI	BM3D	PBNO	EPLL	GID	SAIST	WNNM	WSNM	WIENER	LCDP
Lena	**33.16**	33.05	32.75	32.61	31.74	33.08	33.13	33.09	30.59	31.39	**0.8792**	0.8772	0.8728	0.8691	0.852	0.879	0.879	0.8773	0.8195	0.8773
Monarch	31.18	30.35	29.55	30.48	29.65	30.76	**31.24**	31.16	27.12	29.28	0.9261	0.9179	0.9114	0.9166	0.8984	0.9243	**0.9272**	0.9265	0.8585	0.8855
Cameraman	30.68	30.48	29.61	30.34	29.31	30.45	30.64	30.64	27.28	29.62	0.8775	0.8755	0.8628	**0.8817**	0.8593	0.8775	0.8746	0.875	0.7852	0.8469
Couple	30.78	30.76	30.22	30.54	29.28	30.66	30.77	**30.81**	27.79	29.34	0.8418	**0.8476**	0.8353	0.8399	0.7948	0.8365	0.8416	0.8442	0.7489	0.791
Hill	**30.82**	30.72	30.32	30.49	29.59	30.58	30.79	30.81	28.97	29.66	**0.807**	0.804	0.7907	0.7993	0.7632	0.7977	0.8039	0.8051	0.7456	0.7585
House	34.07	33.77	33.58	32.98	32.81	33.75	**34.2**	33.98	29.72	32.15	0.8736	0.8726	**0.8749**	0.8609	0.8569	0.8689	0.872	0.8723	0.7965	0.8367
Man	30.72	30.59	30.15	30.63	29.59	30.54	**30.73**	30.72	28.97	29.66	0.8357	0.8333	0.8229	**0.8379**	0.7984	0.8316	0.8355	0.836	0.7818	0.7948
Peppers	31.56	31.29	30.55	31.17	30.17	31.32	31.53	**31.62**	28.68	30.18	0.8919	0.8868	0.8775	0.8847	0.8638	0.8888	**0.8954**	0.8908	0.841	0.8559
Straw	27.65	27.07	25.86	26.92	26.63	27.23	27.61	**27.71**	22.03	25.19	0.909	0.8973	0.8577	0.8963	0.8806	0.9095	0.9094	**0.9118**	0.5843	0.8177
Barbara	32.15	31.77	31.06	29.76	30.21	32.1	32.12	**32.16**	25.65	28.83	0.9101	0.9054	0.8957	0.8752	0.8704	**0.9113**	0.909	0.9104	0.7322	0.8321
Boat	**30.95**	30.88	30.39	30.66	29.53	30.84	30.94	**30.95**	28.5	29.63	0.8256	**0.8259**	0.8164	0.8231	0.7834	0.8196	0.8249	0.8246	0.7575	0.7808
Jetplane	32.97	32.53	32.06	32.41	31.48	32.39	32.96	**32.99**	30.5	31.39	**0.9039**	0.9006	0.8962	0.8997	0.8861	0.9024	0.9022	0.9035	0.8451	0.8678
W.bridge	**27.67**	27.27	26.7	27.49	26.49	27.31	27.66	27.64	25.55	26.71	0.801	0.79	0.7619	**0.8117**	0.7461	0.7884	0.7998	0.8002	0.6884	0.7532
Average	**31.1046**	30.8100	30.2153	30.4985	29.7292	30.8469	31.1015	31.0985	27.7960	29.4640	**0.8679**	0.8642	0.8520	0.8612	0.8349	0.8643	0.8673	0.8675	0.7680	0.8193

**Table 2:** Denoising results of different algorithms for given noise level *σ*_*n*_ = 30.

	PSNR/*σ*_*n*_ = 30										SSIM/*σ*_*n*_ = 30									
Image	SAFPI	BM3D	PBNO	EPLL	GID	SAIST	WNNM	WSNM	WIENER	LCDP	SAFPI	BM3D	PBNO	EPLL	GID	SAIST	WNNM	WSNM	WIENER	LCDP
Lena	**31.49**	31.26	31.16	30.78	29.83	30.77	31.38	31.45	28.43	29.41	0.852	0.8449	0.8436	0.8325	0.8068	0.8471	0.8491	**0.8525**	0.7351	0.7702
Monarch	**29.09**	28.36	27.85	28.35	27.6	28.03	28.94	29.02	25.78	27.01	**0.8996**	0.8822	0.8746	0.8789	0.8577	0.8903	0.8949	0.8965	0.81	0.8306
Cameraman	**28.75**	28.63	27.87	28.36	27.84	27.47	**28.75**	28.74	25.74	27.7	**0.838**	0.8373	0.8331	0.8316	0.823	0.8237	0.8369	0.8378	0.7123	0.7912
Couple	28.92	28.86	28.58	28.61	27.15	28.58	**28.98**	28.92	27.2	26.49	0.7928	0.7947	0.7866	0.7831	0.7202	0.7792	**0.7951**	0.7916	0.6932	0.7115
Hill	**29.19**	29.15	28.95	28.9	27.75	28.94	29.18	29.17	27.36	27.91	0.75	**0.7504**	0.7395	0.7418	0.6931	0.7373	0.7421	0.7465	0.6808	0.6875
House	32.61	32.08	31.92	31.22	30.35	31.39	32.46	**32.75**	27.94	29.95	0.8525	0.848	0.8443	0.8338	0.8249	0.8513	0.8514	**0.8543**	0.7246	0.7835
Man	**28.92**	28.86	28.65	28.82	27.82	28.68	**28.92**	**28.92**	27.39	27.79	**0.7803**	0.7802	0.7723	0.7797	0.7378	0.7708	**0.7803**	0.7793	0.7163	0.7207
Peppers	29.55	29.28	28.81	29.16	28.16	28.33	**29.65**	29.51	27.06	27.83	0.8597	0.8505	0.8377	0.8467	0.8189	0.8538	**0.8601**	0.8584	0.7845	0.7912
Straw	**25.54**	24.94	24.7	24.74	24.59	24.74	25.51	25.41	21.73	23.17	0.8526	0.829	0.8213	0.8227	0.8162	0.8483	**0.8533**	0.8503	0.5805	0.7162
Barbara	**30.31**	29.81	29.5	27.56	27.95	30.04	30.28	30.27	24.52	26.48	**0.8802**	0.8687	0.8655	0.8141	0.8129	0.8764	0.8801	0.8792	0.6733	0.7501
Boat	**29.21**	29.11	28.81	28.89	27.66	28.83	29.17	29.09	27.01	27.76	0.777	**0.7795**	0.77	0.7732	0.7286	0.7669	0.7767	0.7755	0.701	0.7158
Jetplane	30.96	27.56	30.21	30.41	29.47	29.35	**31.01**	30.98	28.24	29.25	0.8756	0.8417	0.8361	0.8655	0.8531	0.8731	**0.8763**	0.8754	0.7677	0.8082
W.bridge	**25.79**	25.46	25.22	25.68	24.78	25.43	25.78	25.76	24.72	25.03	0.7123	0.6986	0.6867	**0.7229**	0.6583	0.6888	0.7111	0.7065	0.6555	0.6662
Average	**29.2562**	28.7200	28.6331	28.585754	27.7654	28.5052	29.2315	29.23	26.3392	27.4223	**0.8248**	0.8158	0.8086	0.8097	0.7809	0.8159	0.8240	0.8235	0.7104	0.7495

**Table 3:** Denoising results of different algorithms for given noise level *σ*_*n*_ = 50.

	PSNR/*σ*_*n*_ = 50										SSIM/*σ*_*n*_ = 50									
Image	SAFPI	BM3D	PBNO	EPLL	GID	SAIST	WNNM	WSNM	WIENER	LCDP	SAFPI	BM3D	PBNO	EPLL	GID	SAIST	WNNM	WSNM	WIENER	LCDP
Lena	**29.29**	29.05	28.81	28.42	27.69	29.01	29.24	29.19	24.85	26.98	**0.8116**	0.7994	0.7817	0.7718	0.7663	0.8041	0.8077	0.8087	0.5565	0.6706
Monarch	**26.54**	25.81	25.53	25.77	24.97	26.09	26.37	26.2	23.27	24.14	**0.8482**	0.82	0.798	0.8124	0.7651	0.831	0.8378	0.8383	0.6865	0.7093
Cameraman	**26.63**	26.13	25.71	26.02	25.48	25.94	26.45	26.44	23.14	24.87	**0.7904**	0.7828	0.7526	0.7617	0.7666	0.7766	0.7864	0.7903	0.5547	0.6699
Couple	26.64	26.46	26.3	26.23	24.64	**26.92**	26.63	26.71	23.78	24.85	0.713	0.7068	0.6965	0.6901	0.6139	0.694	0.7118	**0.7165**	0.5531	0.5893
Hill	**27.26**	27.19	27.02	26.95	25.93	27.04	27.22	27.22	24.35	25.78	0.6712	**0.6747**	0.6596	0.6624	0.6162	0.6616	0.6711	0.6684	0.5347	0.5819
House	**30.51**	29.69	29.44	28.76	27.62	29.99	30.28	30.21	24.6	27.08	**0.8284**	0.8122	0.785	0.7845	0.7715	0.8236	0.8208	0.8273	0.5535	0.6846
Man	26.9	26.8	26.72	26.72	25.83	26.67	**26.91**	26.88	24.28	25.65	**0.7101**	0.7056	0.6939	0.6976	0.6631	0.6977	0.7099	0.7081	0.5593	0.6135
Peppers	26.96	26.68	26.46	26.62	25.6	26.6	26.94	**27.07**	24.14	24.98	0.8038	0.7936	0.7627	0.7832	0.7602	0.7999	0.7991	**0.8088**	0.6352	0.6717
Straw	**22.99**	22.4	22.81	22	21.98	22.65	22.91	**22.99**	20.69	20.98	0.7389	0.6881	0.7328	0.6489	0.6768	0.7258	0.7349	**0.7435**	0.5433	0.5601
Barbara	**27.94**	27.22	26.95	24.82	25.17	27.49	27.83	27.81	22.45	23.86	**0.8229**	0.7946	0.7849	0.7017	0.7013	0.8033	0.8212	0.8225	0.5315	0.6084
Boat	26.86	26.78	26.67	26.65	25.59	26.63	26.85	**26.92**	24.05	25.33	0.7038	0.7053	0.6936	0.695	0.6538	0.6921	**0.7066**	0.7063	0.5504	0.6022
Jetplane	28.57	25.1	27.77	27.88	26.91	28.25	28.56	**28.58**	24.78	26.57	0.8378	0.8269	0.7954	0.8059	0.8062	0.8314	0.8307	**0.84**	0.5858	0.6983
W.bridge	23.85	23.57	23.49	23.69	22.88	23.49	**23.87**	23.81	22.79	23.13	0.5874	0.5715	0.5726	**0.5901**	0.5289	0.5556	0.5893	0.5863	0.5609	0.5455
Average	**26.9954**	26.3754	26.4369	26.1945	25.4069	26.6746	26.9277	26.9254	23.6285	24.9385	**0.7590**	0.7447	0.7315	0.7235	0.6992	0.7459	0.7559	0.7588	0.5696	0.6312

**Table 4:** Denoising results of different algorithms for given noise level *σ*_*n*_ = 60.

	PSNR/*σ*_*n*_ = 60										SSIM/*σ*_*n*_ = 60									
Image	SAFPI	BM3D	PBNO	EPLL	GID	SAIST	WNNM	WSNM	WIENER	LCDP	SAFPI	BM3D	PBNO	EPLL	GID	SAIST	WNNM	WSNM	WIENER	LCDP
Lena	**28.46**	28.27	27.92	27.59	26.91	28	28.39	28.45	23.41	26.06	**0.7937**	0.7795	0.7523	0.746	0.7446	0.7895	0.7865	0.793	0.4828	0.6176
Monarch	**25.49**	24.97	24.64	24.85	24.15	24.94	25.44	25.45	22.26	23.09	**0.819**	0.7926	0.7571	0.782	0.766	0.7966	0.8105	0.8154	0.6256	0.6621
Cameraman	**25.78**	25.31	24.98	25.2	24.5	25.15	25.63	25.63	22.03	23.82	**0.7741**	0.7625	0.7237	0.7342	0.7348	0.7527	0.7637	0.7714	0.4868	0.6184
Couple	**25.87**	25.66	25.43	25.4	24.01	24.98	25.79	25.82	22.63	24.02	**0.6854**	0.6715	0.6537	0.651	0.5824	0.6679	0.6754	0.6798	0.4896	0.5417
Hill	**26.54**	**26.54**	26.27	26.27	25.32	26.39	26.52	**26.54**	23.05	25.08	0.646	**0.647**	0.6255	0.632	0.5892	0.6429	0.6418	0.644	0.4705	0.5423
House	**29.66**	28.73	28.62	27.84	26.66	28.88	29.38	29.39	23.16	26	**0.8172**	0.7941	0.7649	0.7604	0.7484	0.8049	0.8044	0.8101	0.4842	0.6262
Man	**26.25**	26.13	26	26	25.14	25.78	26.22	26.22	22.96	24.86	0.6829	0.6786	0.662	0.6667	0.6391	0.677	0.683	**0.6854**	0.4894	0.5672
Peppers	**26.11**	25.81	25.66	25.67	24.64	25.63	26.08	26.03	22.86	23.95	**0.7835**	0.7698	0.7371	0.7557	0.728	0.765	0.7744	0.7785	0.5724	0.6232
Straw	22.12	21.63	22.01	21.06	20.93	**22.13**	21.99	22.05	20.08	20.37	0.6858	0.6285	0.6815	0.5614	0.5904	**0.6905**	0.6681	0.6834	0.5234	0.5118
Barbara	26.95	26.28	26.08	23.87	24.19	26.4	26.88	**26.96**	21.53	23.07	0.7922	0.7589	0.7513	0.6538	0.6529	0.7833	0.7898	**0.7961**	0.4658	0.554
Boat	**26.17**	26.02	25.94	25.84	24.68	25.52	26.07	26.09	22.85	24.51	0.6797	0.6767	0.6643	0.6625	0.6239	0.6715	0.6777	**0.6816**	0.4904	0.5595
Jetplane	27.71	27.32	26.98	26.97	25.82	26.64	27.7	**27.74**	23.39	25.67	**0.822**	0.8075	0.772	0.779	0.7745	0.8153	0.8105	0.819	0.516	0.6502
W.bridge	23.2	23.02	22.9	23.08	22.19	22.85	**23.23**	23.21	21.81	22.49	**0.5464**	0.5339	0.5311	0.5443	0.4736	0.5328	0.5426	0.5412	0.513	0.5025
Average	**26.1777**	25.8223	25.6485	25.3569	24.5492	25.6377	26.1015	26.1215	22.4630	24.0762	**0.7329**	0.7155	0.6982	0.6869	0.6652	0.7223	0.7253	0.7307	0.5085	0.5828

**Table 5:** Denoising results of different algorithms for given noise level *σ*_*n*_ = 75.

	PSNR/*σ*_*n*_ = 75										SSIM/*σ*_*n*_ = 75									
Image	SAFPI	BM3D	PBNO	EPLL	GID	SAIST	WNNM	WSNM	WIENER	LCDP	SAFPI	BM3D	PBNO	EPLL	GID	SAIST	WNNM	WSNM	WIENER	LCDP
Lena	**27.55**	27.25	27	26.57	25.96	26.97	27.53	27.34	21.61	24.96	0.7662	0.7516	0.7238	0.7101	0.7126	0.7642	0.767	**0.7707**	0.3982	0.5617
Monarch	24.14	23.9	23.62	23.71	22.77	23.95	**24.28**	23.95	20.72	22.08	0.7753	0.7557	0.7153	0.7395	0.6956	0.7639	**0.7788**	0.7761	0.5437	0.6114
Cameraman	**24.69**	24.32	24.01	24.19	23.26	24.27	24.56	24.5	20.57	22.76	0.7459	0.734	0.6766	0.6955	0.677	0.7296	0.734	**0.7486**	0.3889	0.539
Couple	**25.01**	24.7	24.51	24.44	23.27	24.17	24.87	24.89	21.03	23.16	0.6385	0.626	0.6086	0.6017	0.5454	0.6182	0.637	**0.6393**	0.4116	0.4882
Hill	**25.77**	25.67	25.45	25.45	24.62	25.5	**25.77**	25.71	21.34	24.08	**0.6185**	0.6118	0.5901	0.5936	0.5599	0.606	0.6119	0.6083	0.391	0.4814
House	**28.39**	27.5	27.15	26.68	25.16	27.9	28.18	28.01	21.36	24.85	0.7957	0.7645	0.7094	0.7251	0.6951	0.7872	0.7842	**0.7987**	0.3905	0.563
Man	**25.4**	25.31	25.11	25.14	24.38	25.06	**25.4**	25.35	21.3	23.83	0.6494	0.6445	0.6195	0.6274	0.604	0.6424	**0.6538**	0.6518	0.4085	0.5048
Peppers	24.93	24.73	24.55	24.56	23.34	24.68	24.98	**25**	21.11	22.68	0.7524	0.7368	0.69	0.7198	0.6934	0.7395	0.7426	**0.7582**	0.4735	0.5581
Straw	21.11	20.72	21.04	20.07	19.55	21.08	21.13	**21.15**	19.15	19.65	0.6282	0.5462	0.6038	0.4567	0.4458	0.5959	0.6077	**0.6289**	0.4778	0.4401
Barbara	25.85	25.12	24.94	22.94	23.06	25.35	**25.9**	25.83	20.28	22.21	0.7489	0.7112	0.7006	0.6002	0.5833	0.7369	**0.7576**	0.7574	0.3942	0.491
Boat	25.13	25.14	24.85	24.88	23.81	24.8	**25.19**	25.16	21.22	23.47	0.642	0.641	0.6143	0.6212	0.5877	0.6369	**0.6461**	0.6451	0.4092	0.499
Jetplane	26.7	26.31	25.83	25.83	24.69	25.82	**26.72**	26.59	21.58	24.56	0.7924	0.7812	0.729	0.7414	0.7582	0.792	0.7895	**0.8055**	0.423	0.588
W.bridge	22.55	22.4	22.26	22.39	21.52	22.07	**22.57**	22.44	20.53	21.69	**0.5017**	0.4905	0.485	0.4939	0.4329	0.4725	0.5007	0.4889	.4461	0.448
Average	**25.1708**	24.8515	24.6400	24.3731	23.4915	24.74	25.16	25.0708	20.9077	23.0754	0.6965	0.6765	0.6512	0.6405	0.6147	0.6835	0.6931	**0.6983**	0.4274	0.5211

**Table 6:** Average denoising result of different algorithms for given noise level *σ*_*n*_ = 100.

	PSNR/*σ*_*n*_ = 100										SSIM/*σ*_*n*_ = 100									
Image	SAFPI	BM3D	PBNO	EPLL	GID	SAIST	WNNM	WSNM	WIENER	LCDP	SAFPI	BM3D	PBNO	EPLL	GID	SAIST	WNNM	WSNM	WIENER	LCDP
Lena	**26.33**	25.95	25.6	25.3	24.64	25.81	26.25	26.22	19.23	23.39	0.7317	0.709	0.6672	0.6577	0.6604	0.7275	0.7294	**0.7445**	0.299	0.472
Monarch	22.84	22.51	22.19	22.23	20.83	22.63	**22.94**	22.71	18.82	20.61	0.724	0.7021	0.6415	0.6771	0.6344	0.7156	**0.7337**	**0.7337**	0.4474	0.5223
Cameraman	**23.42**	23.08	22.65	22.85	21.72	23.08	23.35	23.05	18.53	21.43	0.7024	0.6928	0.5868	0.6351	0.6471	0.6963	0.691	**0.7072**	0.2938	0.4487
Couple	23.59	23.51	23.28	23.32	22.38	23.01	**23.63**	23.6	18.94	22.06	0.5653	0.5665	0.5395	0.5383	0.497	0.5539	0.5717	**0.5832**	0.3185	0.4164
Hill	24.61	24.58	24.33	24.42	23.79	24.29	24.7	**24.74**	19.12	22.84	0.564	0.565	0.5355	0.5421	0.5196	0.5566	0.566	**0.5726**	0.2957	0.4088
House	**27.06**	25.87	25.42	25.19	23.59	26.45	26.65	26.4	19.07	23.31	0.761	0.7203	0.6408	0.6695	0.634	0.7566	0.7499	**0.764**	0.3013	0.491
Man	**24.39**	24.22	23.98	24.07	23.33	23.98	24.28	24.2	19.02	22.57	0.6076	0.5978	0.5626	0.5729	0.5504	0.5978	0.6084	**0.6144**	0.3078	0.4316
Peppers	23.56	23.39	23.03	23.08	21.61	23.35	**23.67**	23.32	19.03	21.13	**0.7047**	0.6881	0.6164	0.6653	0.6254	0.7037	0.7002	0.7014	0.3719	0.4729
Straw	19.84	19.58	**19.86**	19.01	18.41	19.54	19.77	**19.86**	17.71	18.81	0.4642	0.4224	0.4853	0.3295	0.3121	0.4106	0.454	**0.5093**	0.4206	0.3701
Barbara	**24.5**	23.62	23.42	22.14	21.76	23.98	24.39	24.43	18.38	21.09	0.6974	0.643	0.6199	0.5463	0.5368	0.6806	0.6925	**0.7069**	0.2998	0.4077
Boat	24	23.97	23.62	23.71	22.74	23.67	**24.09**	24.07	19.03	22.15	0.5989	0.5936	0.5557	0.5653	0.5352	0.5911	0.6002	**0.6109**	0.3166	0.4209
Jetplane	25.39	22.11	24.31	24.35	23.28	24.55	**25.46**	25.3	19.17	23.01	0.7542	0.7442	0.6588	0.6836	0.6927	0.7562	0.7521	**0.7724**	0.3167	0.4957
W.bridge	**21.76**	21.6	21.42	21.58	20.74	21.21	21.69	21.6	18.57	20.77	**0.4503**	0.4398	0.4287	0.4364	0.3866	0.4117	0.441	0.4381	0.3586	0.3902
Average	**23.9454**	23.3838	23.3162	23.1731	22.2169	23.5038	23.9131	23.8077	18.8169	21.7823	0.6404	0.6219	0.5799	0.5784	0.5563	0.6276	0.6377	**0.6507**	0.3344	0.4422

**Table 7:** Comparison of average PNSR with different methods.

*σ*_*n*_	Average PSNR									
	SAFPI	BM3D	PBNO	EPLL	GID	SAIST	WNNM	WSNM	WIENER	LCDP
20	**31.1046**	30.8100	30.2153	30.4985	29.7292	30.8469	31.1015	31.0985	27.7960	29.4640
30	**29.2562**	28.7200	28.6331	28.585754	27.7654	28.5052	29.2315	29.23	26.3392	27.4223
50	**26.9954**	26.3754	26.4369	26.1945	25.4069	26.6746	26.9277	26.9254	23.6285	24.9385
60	**26.1777**	25.8223	25.6485	25.3569	24.5492	25.6377	26.1015	26.1215	22.4630	24.0762
75	**25.1708**	24.8515	24.6400	24.3731	23.4915	24.74	25.16	25.0708	20.9077	23.0754
100	**23.9454**	23.3838	23.3162	23.1731	22.2169	23.5038	23.9131	23.8077	18.8169	21.7823

**Fig 4:**
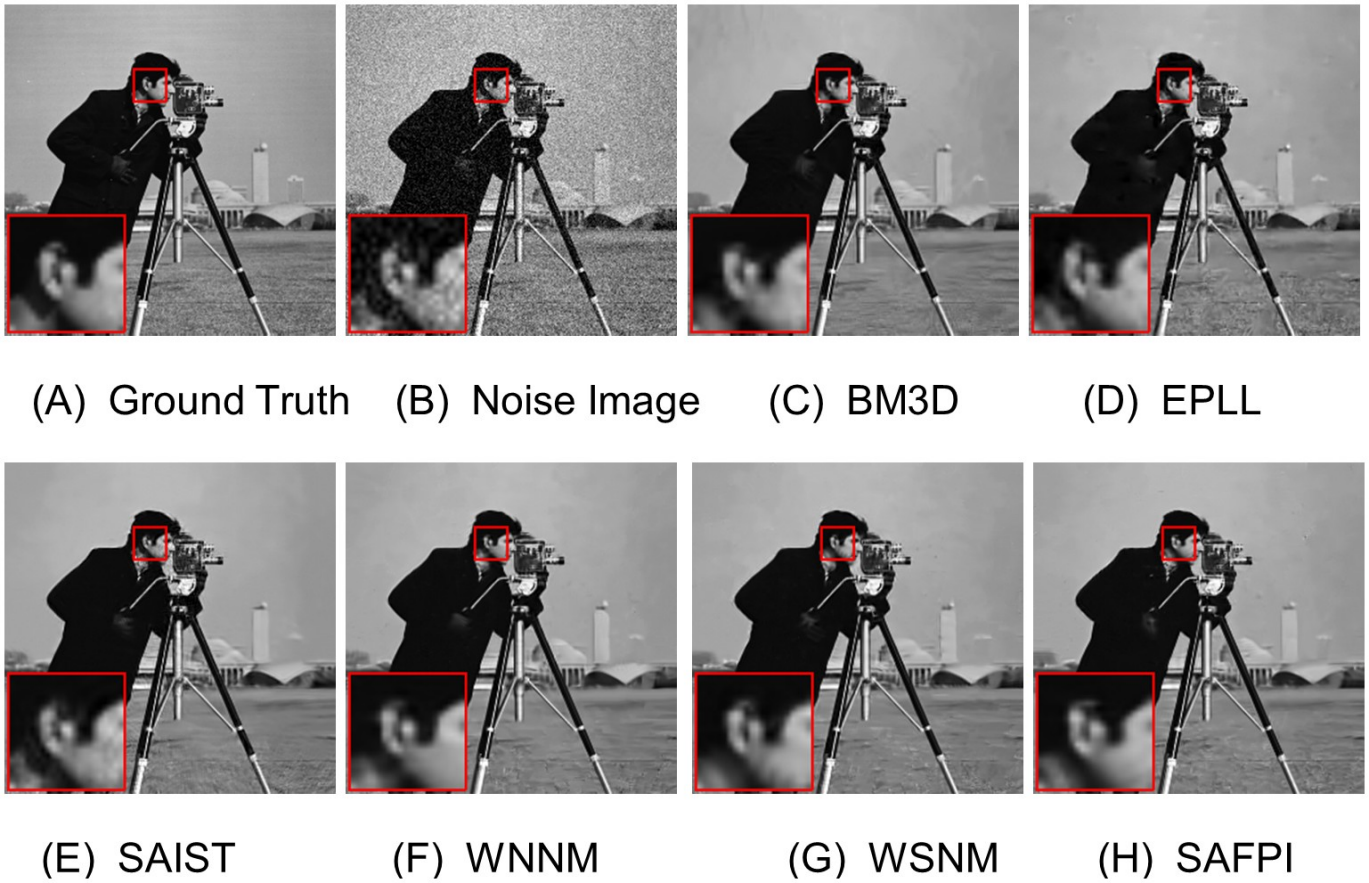
Denoising results on image Cameraman by different methods (noise level *σ*_*n*_ = 20). (A) Ground Truth (B) Noisy Image (C) BM3D, PSNR = 30.48 (D) EPLL, PSNR = 30.34 (E) SAIST, PSNR = 30.45 (F) WNNM, PSNR = 30.64 (G) WSNM, PSNR = 30.64 (H) SAFPI, PSNR = 30.68.

**Fig 5:**
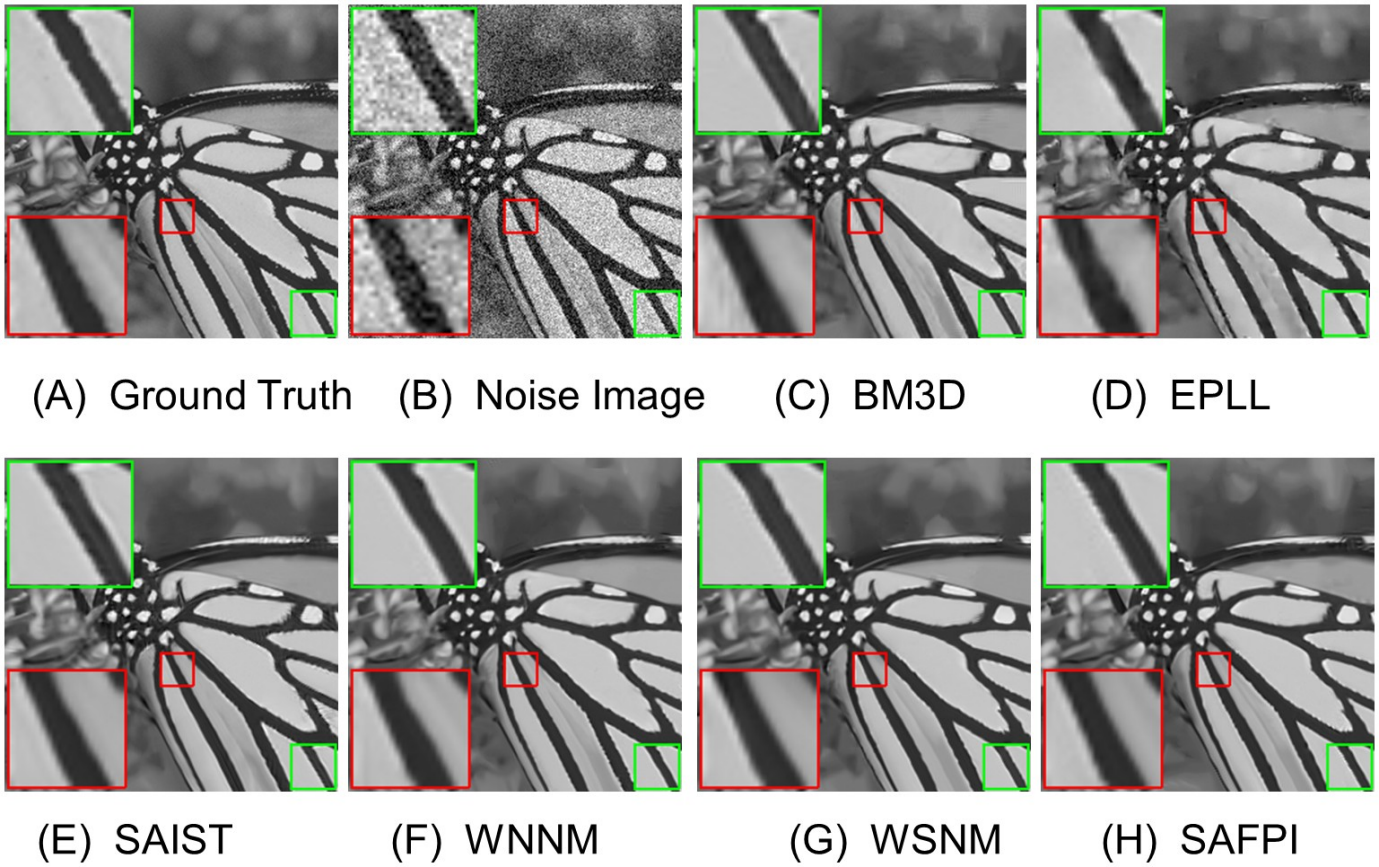
Denoising results on image Monarch by different methods (noise level *σ*_*n*_ = 30). (A) Ground Truth (B) Noisy Image (C) BM3D, PSNR = 28.36 (D) EPLL, PSNR = 28.35 (E) SAIST, PSNR = 28.03 (F) WNNM, PSNR = 28.94 (G) WSNM, PSNR = 29.02 (H) SAFPI, PSNR = 29.09.

**Fig 6:**
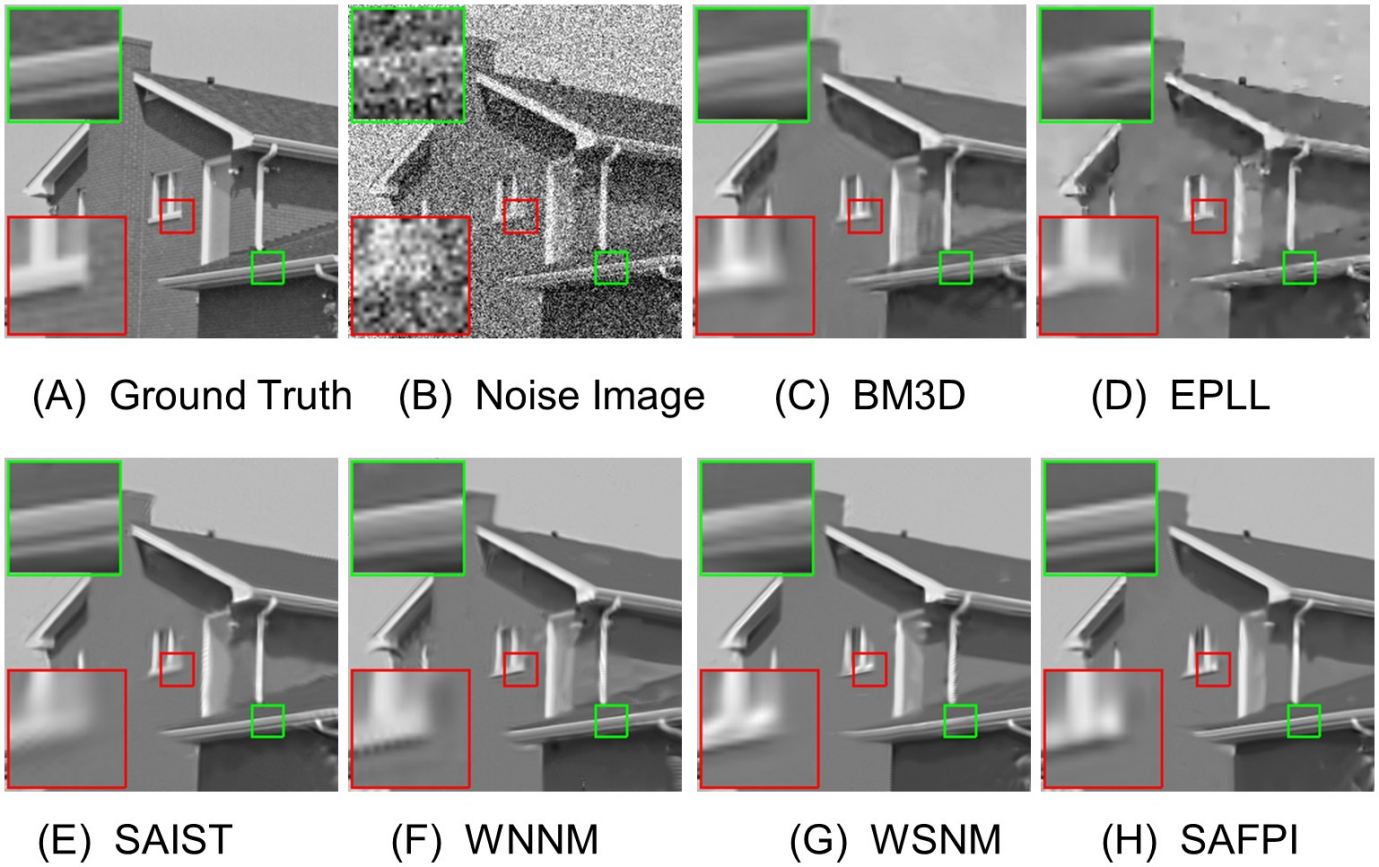
Denoising results on image House by different methods (noise level *σ*_*n*_ = 50). (A) Ground Truth (B) Noisy Image (C) BM3D, PSNR = 29.69 (D) EPLL, PSNR = 28.76 (E) SAIST, PSNR = 29.99 (F) WNNM, PSNR = 30.28 (G) WSNM, PSNR = 30.21 (H) SAFPI, PSNR = 30.51.

**Fig 7:**
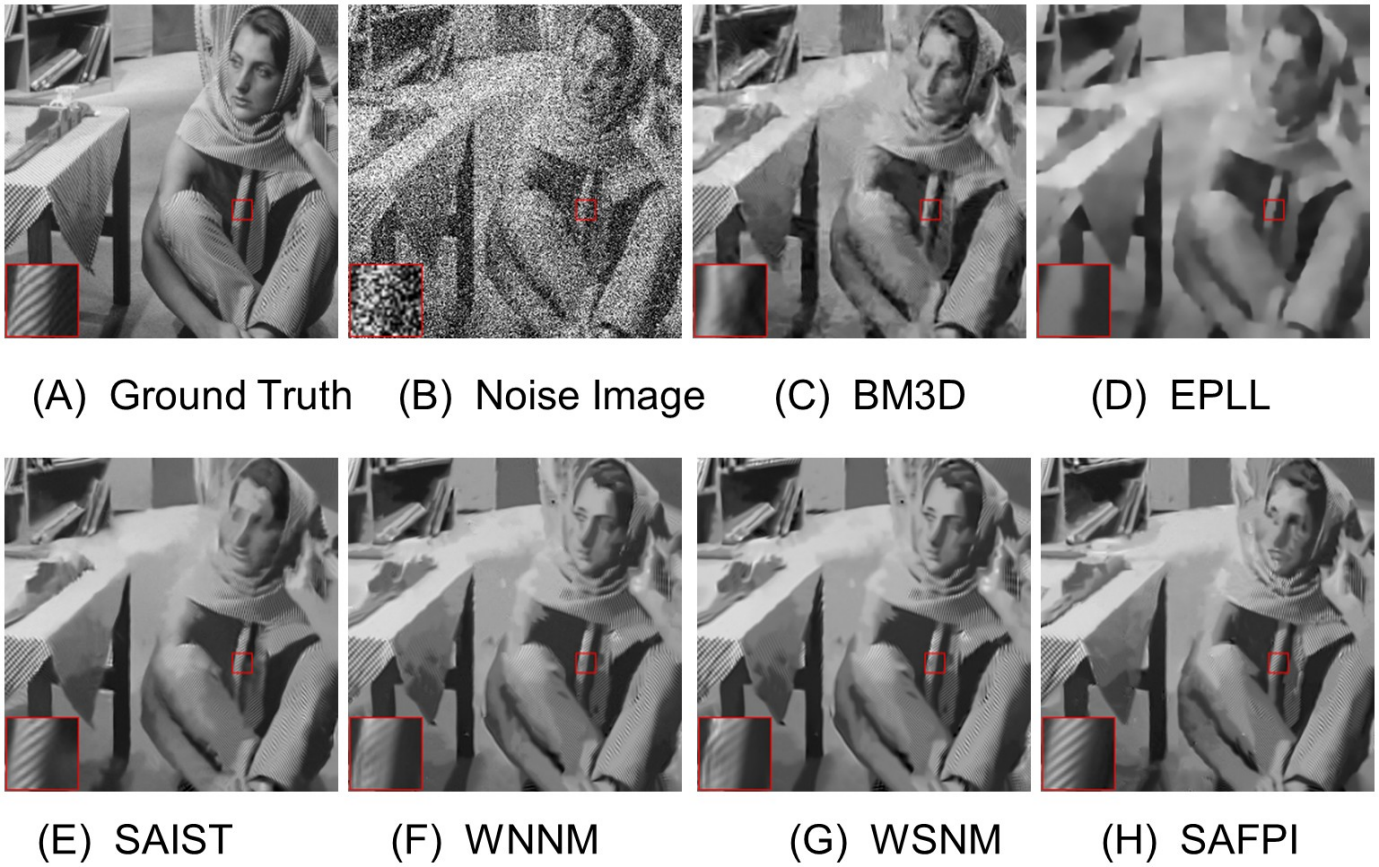
Denoising results on image Barbara by different methods (noise level *σ*_*n*_ = 100). (A) Ground Truth (B) Noisy Image (C) BM3D, PSNR = 23.62 (D) EPLL,PSNR = 22.14 (E) SAIST, PSNR = 23.98 (F) WNNM, PSNR = 24.39 (G) WSNM, PSNR = 24.43 (H) SAFPI, PSNR = 24.5.

For visual quality, some comparative images are shown in Figs 4, 5, 6 and 7. As shown in Fig 4, our algorithm resumed the structure of the ear (which is magnified in the highlighted red window) better than other algorithms. When the noise level is very high, as shown in the zoom-in window in Fig 7, our algorithm could reconstruct clear texture structures, while the competing methods get more blurred textures. Other visual improvements can be seen in Figs 5 and 6. Sometimes the variation of noise is too big and too small in the same image (in different parts of the image). To demonstrate our method, we randomly selected two small pieces from the given image. Although their local noise level would be different, our algorithm always gets the best visual texture. Now we could conclude that the proposed SAFPI algorithm can display excellent denoising performance, producing good visual effect and rebuilding better textures.

If noise is non-Gaussian, one popular method is to transform the non-Gaussian noise into a more tractable Gaussian model such as the generalized Anscombe transformation (GAT) [43, 44]. In this paper, we deal with non-Gaussian noise using the proposed algorithms. In the first experiment, we assumed the noise was a mix of Gaussian noise (*σ*_*n*_ = 20, 50, 100) and speckle noise (the density is *d* = 1*10^−3^). Then we used the SAFPI algorithm directly to remove the noise. We randomly selecteded six images (Lena, Monarch, Barbara, Cameraman, House, Peppers) on Fig 1 for experimental verification and compared with some excellent denoising algorithms which has been mentioned in the previous experiments. The results are shown on Table 8. In the second experiment, we assumed the noise was mixed Poisson-Gaussian noise. Then we transformed the Poisson-Gaussian hybrid noise into an approximate Gaussian noise using the GAT [43] algorithm and obtained the repaired images by using the proposed denoising algorithm and the exact unbiased inverse GAT [44]. We used Lena and C.man as the test images and set eight different peak values to be (1, 2, 5, 10, 20, 30, 60, 120). The Poisson-Gaussian noise were set to be *σ* = peak/10. We compared with BM3D, SAIST, WNNM and recorded the average PSNR and kurtosis parameters of these two images. The results are shown on Table 9. All bold numbers represent the best evaluation index values. From Table 8, we can see when the standard deviation is not big (*σ*_*n*_ = 20, 50), our proposed algorithm almost achieved the best values of all three quality evaluation indicators, and obtained the best PSNR values on all of hybrid noises experiments. From Table 9, it can be seen that the SAFPI algorithm almost get the highest averaged PSNR value under all peaks experiments. In most cases, our proposed algorithm obtained relatively good kurtosis metrics and optimal PSNR value, which is 0.2 to 0.3dB higher than the BM3D algorithm and about 0.1dB to 1.11dB higher than WNNM.

**Table 8:** Average denoising results of different algorithms for Speckle-Gaussian noise.

	Speckle-Gaussian(*σ*_*n*_ = 20)			Speckle-Gaussian(*σ*_*n*_ = 50)			Speckle-Gaussian(*σ*_*n*_ = 100)		
	Kurtosis	PSNR	SSIM	Kurtosis	PSNR	SSIM	Kurtosis	PSNR	SSIM
NOISES	2.3067	21.9183	0.4126	2.7447	14.1117	0.195	2.9562	8.1233	0.0726
BM3D	2.0927	31.5617	0.8782	2.1057	27.4067	0.7716	**2.1343**	24.0083	0.6427
EPLL	2.1027	29.1417	0.8268	2.1278	25.8683	0.7079	2.1423	24.7567	0.6572
SAIST	2.0897	31.81	0.8845	2.105	27.53	0.7811	2.1733	24.2833	0.6545
WNNM	2.0762	32.0383	0.8843	**2.0978**	27.82	0.7887	2.1412	24.4817	0.668
WSNM	2.0743	32.0117	0.8842	2.1	27.8783	0.7921	2.1872	24.3933	**0.6807**
SAFPI	**2.0739**	**32.0433**	**0.8846**	2.112	**27.88**	**0.7925**	2.1778	**24.5**	0.6729

**Table 9:** Average denoising results of different algorithms for Poisson-Gaussian noise.

Image	Peak	*σ*_*n*_	Noisy	PSNR				Kurtosis			
				GAT+SAFPI	GAT+BM3D	GAT+SAIST	GAT+WNNM	GAT+SAFPI	GAT+BM3D	GAT+SAIST	GAT+WNNM
C.man and Lena	1	0.1	3.05	20.485	**21.44**	18.655	19.37	**2.198**	2.2685	2.3745	2.2425
	2	0.2	5.97	**23.085**	**23.085**	22.02	22.83	**2.152**	2.1695	2.1855	2.157
	5	0.5	9.69	**25.41**	25.035	24.66	24.335	2.0965	2.13	2.131	**2.0885**
	10	1	12.32	**26.795**	26.51	26.265	26.12	2.1685	2.1765	**2.163**	2.1675
	20	2	14.64	**28.075**	27.83	27.57	27.845	2.1495	2.3745	2.1435	**2.13**
	30	3	15.80	**28.695**	28.43	28.26	28.57	2.136	2.1855	2.1437	**2.1275**
	60	6	17.43	**29.46**	29.205	29.085	29.385	2.1385	2.131	2.1365	**2.125**
	120	12	18.54	**30.015**	29.73	29.67	29.995	2.152	2.163	2.145	**2.1345**

Finally, we bravely attempted to discuss the complexity of SAFPI algorithm. We assume each patch size is *A* * *A*, where *A* represents the length or width of each block, and *k* is the number of similar patches in each structural group **y**_*i*_. Now calculating SVD (step 4 in Algorithm 1) needs $O(\min\left(A^{2}*K^{2},\;A^{4}*K\right))$ flops in each iteration. And it also costs O(K) to compute the singular values in step 6. Next since the image **y**^(*k*+1)^ can be divided into *N* blocks in step 2, then it needs $i*N*O(\min\left(A^{2}*K^{2},\;A^{4}*K\right)+K)$ flops, where *i* is the number of iterations in Algorithm 1. Then we recorded the execution times of several excellent denoising algorithms spent on the above experiments with the standard deviation *σ*_*n*_ of the white Gaussian noise to be 20: SAFPI 4843.339s, WSNM 5453.311s, WNNM 4410.991s, SAIST 923.9837s, BM3D 17.6242s and EPLL 1550.607s. Our algorithm did not take much longer time while maintaining the best denoising results.

## 4 Conclusions

In this paper, a fixed-point iteration scheme was developed for sparse optimization in *ℓ*_*p*_ space with *p* ∈ (0, 1] by using proximal operator. We showed that group sparse coding was equivalent to Schatten-*p* norm minimization problem, and thus the sparse coefficient of each group were measured by estimating the singular values of each group. When analyzing the optimal value of power *p*, we can find that the optimal value of Schatten *p*-norm is related to the noise level. As the noise level increases, the optimal value of *p* decreases gradually. And if the noise reaches a high level, the optimal value of *p* will be close to 1. The developed SAFPI algorithm can obtain higher PSNR indices and is able to retain promising texture structure information and visual quality. The methods developed in this paper leads to a better image denoising compared to other competing denoising algorithms. There are several future research directions. We are further exploring other non-convex optimization strategies for more effective convergence and further improvement. The convolutional neural networks(CNN) based denoising methods become more and more popular now and we will investigate CNN architectures for the denoising of images in the future.

## 5 Appendix

**Proof 9 (Proof of Theorem 1)** *Let* **D** = **U** *and* **A** = Σ**V**^*T*^ *in* Eq (1), *where* $\Sigma =\text{diag}\left\{\varepsilon_{1},\ldots ,\varepsilon_{K}\right\}\left(K=\min\left\{m,n\right\}\right)\in R^{K\times K}$ *is a diagonal matrix and each column of* **V** *in* $R^{m\times K}$ *is decomposed of* **v**_*i*_ = (*α*^*i*^)^*T*^/*ε*_*i*_. *Then we have*

$$\begin{array}{c} \left(U,\Sigma ,V\right)=\arg\min_{U,\Sigma ,V}\frac{1}{2}||Y-U\Sigma V^{T}{\left|\right|}_{F}^{2}+{\lambda ||A||}_{p,2}^{p}. \end{array}$$ (9)

*Let σ*_*i*_ *denotes the standard deviation of the sparse coefficients α*^*i*^ *in the i-th column, then the sum of standard deviations associated with sparse coefficient vector in each column is*

$$\begin{array}{c} \alpha_{i,1}^{2}+\cdots +\alpha_{i,m}^{2}=m\sigma_{i}^{2}. \end{array}$$ (10)

*And then it is not hard to see*

$$\begin{array}{c} {\left||A|\right|}_{p,2}=\sqrt[{p}]{\sum_{i=1}^{K}{\left(\alpha_{i,1}^{2}+\cdots +\alpha_{i,m}^{2}\right)}^{p/2}}=\sqrt{m}\sqrt[{p}]{\sigma_{1}^{p}+\cdots +\sigma_{K}^{p}}. \end{array}$$ (11)

*Using* Eq (10) *and the unitary property of* **V**, *we have*

$$\begin{array}{c} \sigma_{i}^{2}=\frac{1}{m}\left|\right|\alpha^{i}{\left|\right|}_{2}^{2}=\frac{1}{m}\left|\right|\varepsilon_{i}v_{i}^{T}{\left|\right|}_{2}^{2}=\frac{\varepsilon_{i}^{2}}{m}. \end{array}$$ (12)

*Then it is ready to see*

$$\begin{array}{c} {\left||A|\right|}_{p,2}=\sqrt[{p}]{\varepsilon_{1}^{p}+\cdots +\varepsilon_{K}^{p}}. \end{array}$$ (13)

*By substituting* Eq (13) *into* Eq (9) *we could obtain*

$$\begin{array}{cc} \left(U,\Sigma ,V\right) & =\arg\min_{U,\Sigma ,V}\frac{1}{2}||Y-U\Sigma V^{T}{\left|\right|}_{F}^{2}+\lambda \sum_{i=1}^{K}\varepsilon_{i}^{p}\\ & =\arg\min_{X=U\Sigma V^{T}}\frac{1}{2}||Y-{X||}_{F}^{2}+{\lambda ||X||}_{S_{p}}^{p}, \end{array}$$ (14)

*which appears to be better approximation to the rank function by using the Schatten-p quasi-norm*.

**Proof 10 (Proof of Lemma 6)** *Let ϕ* = |*w*|^*p*^, *by using subdifferential and Corollary 2.59 of* [27], *one has*

$$\begin{array}{c} {\text{Prox}}_{\lambda }∥\cdot {∥}_{p}\left(x\right)\subseteq {\left(I+\lambda \partial \phi \right)}^{-1}\left(x\right). \end{array}$$

*If ϕ is convex, this is an equality*.

*If we further define* $\Pi \left(w\right)={\lambda |w|}^{p}+\frac{w^{2}}{2}$, *since*

$$\begin{array}{c} \arg\min_{w}{\{\left(w-x\right)}^{2}+{2\lambda |w|}^{p}\}=\arg\min_{w}\left\{\Pi \left(w\right)-wx\right\}, \end{array}$$ (15)

*it is ready to see*

$$\begin{array}{c} {\text{Prox}}_{\lambda }∥\cdot {∥}_{p}\left(x\right)=\Pi^{*}\left(x\right). \end{array}$$ (16)

*For* Π*(*x*) *is the pointwise minimum of the collection of affine functions, we know it is closed and convex. Thus* **Prox**_λ_ ∥ ⋅∥_*p*_(*x*) *is also closed and convex*.

*But it is easy to see* Π*(*x*) *is a discontinues mapping, so generally there is no closed form expression for it*.

**Proof 11 (Proof of Theorem 8)** *For* 0 < *p* < 1, *let ϕ*(*w*) = |*w*|^*p*^, *we have* ∂*ϕ*(*w*) = ∅ *when w* = 0.

*In order to overcome the singularity of* (|*w*|^*p*^)′ = *pw*/|*w*|^2−*p*^ *near w* = 0, *following Section 4 of* [28], *we consider for* 0 < *ϵ* << 1 *the approximation*

$$\begin{array}{c} \partial \phi \left(w\right)\approx \frac{pw}{\max(\epsilon^{2-p},{\left|w\right|}^{2-p})}. \end{array}$$

*It is important to observe that* **Prox**_λ_ ∥⋅∥_*p*_(*x*) = 0 *if*

$$\begin{array}{c} {\left|x\right|}^{2}\leq {\left(w-x\right)}^{2}+2\lambda w^{p}, \end{array}$$ (17)

*which is equivalent to*

$$\begin{array}{c} x\leq \min\frac{w+2\lambda w^{p-1}}{2}=\frac{2-p}{2(1-p)}{\left(2\lambda \left(1-p\right)\right)}^{\frac{1}{2-p}}, \end{array}$$

*equality obtained when* $w=\sqrt[{p-2}]{2\lambda (1-p)}$. *Otherwise, the necessary optimality condition is given by*

$$\begin{array}{c} w-x+\frac{\lambda p}{\max(\epsilon^{2-p},|w{|}^{2-p})}w=0. \end{array}$$ (18)

*To solve* Eq (18) *for nonnegative w, let*

$$\begin{array}{c} g\left(w\right)=\left|x\right|-\frac{\lambda p}{\max(\epsilon^{2-p},|w{|}^{2-p})}w, \end{array}$$

*we consider the iteration*

$$\begin{array}{c} w^{\left(n\right)}=g\left(w^{\left(n-1\right)}\right)=\left|x\right|-\frac{\lambda p}{\max(\epsilon^{2-p},|w^{\left(n-1\right)}{|}^{2-p})}w^{\left(n-1\right)}. \end{array}$$

*One can easily see that*

$$\begin{array}{c} |g^{\prime }\left(w\right)|=\lambda p\left(1-p\right)w^{p-2}<1, \end{array}$$

*if and only if* $\left|w\right|\geq \sqrt[{2-p}]{\lambda p(1-p)}$. *Notice that the first and second order derivatives of f*(*w*) = (*w* − *x*)^2^ + 2λ|*w*|^*p*^ *are*

$$\begin{array}{cc} f^{\prime }\left(w\right) & =w-x+\lambda pw^{p-1} \end{array}$$

$$\begin{array}{cc} f^{\prime \prime }\left(w\right) & =1+\lambda p\left(p-1\right)w^{p-2}, \end{array}$$

*and one can easily verify that f*(*w*) *is concave in the range of* $\left(0,\sqrt[{2-p}]{\lambda p(1-p)}\right)$, *and is convex in the range of* $\left(\sqrt[{2-p}]{\lambda p(1-p)},\infty \right)$. *By using the Contraction Mapping Theorem (Theorem 1.5 on page 11 of* [30]), *we have*

$$\begin{array}{c} w^{\left(1\right)}=\left|x\right|-{\lambda p|x|}^{p-1},w^{\left(2\right)}=\left|x\right|-\lambda p(|x|-{\lambda p|x|}^{p-1}{)}^{p-1},\ldots . \end{array}$$

*The iteration will eventually converge to a fixed-point, which is the root w* = *g*(*w*) in the interval $\left(\sqrt[{2-p}]{\lambda p(1-p)},\infty \right)$.

*Moreover, by noting that the derivative of x*(*w*) = *w* + λ*pw*^*p*−1^ *is*

$$\begin{array}{cc} \dot{x}\left(w\right) & =1+\lambda p\left(p-1\right)w^{p-2}, \end{array}$$

*by solving* $\dot{x}\left(w\right)=0$, *we have*

$$\begin{array}{c} \bar{w}=\sqrt[{2-p}]{\lambda p(1-p)}, \end{array}$$

*then*

$$\begin{array}{c} \min x\left(w\right)=x_{\bar{w}}=\bar{w}+\lambda p{\bar{w}}^{p-1}={\left(\lambda p\left(1-p\right)\right)}^{\frac{1}{2-p}}+\lambda p{\left(\lambda p\left(1-p\right)\right)}^{\frac{p-1}{2-p}}, \end{array}$$

*and* $\left|w\right|\geq \sqrt[{2-p}]{\lambda p(1-p)}\Rightarrow \left|x\right|>{\left(\lambda p\left(1-p\right)\right)}^{\frac{1}{2-p}}+\lambda p{\left(\lambda p\left(1-p\right)\right)}^{\frac{p-1}{2-p}}$. *Combined with* (17), *we denote*

$$\begin{array}{c} {ℵ}_{\lambda ,p}=\max\{\frac{2-p}{2(1-p)}{\left(2\lambda \left(1-p\right)\right)}^{\frac{1}{2-p}},\frac{1}{2}{\left(\lambda p\left(1-p\right)\right)}^{\frac{1}{2-p}}+\lambda {\left(\lambda p\left(1-p\right)\right)}^{\frac{p-1}{2-p}}\}. \end{array}$$

*Thus the proof is completed*.
